# Spec-LAMP: Robust Spectre Attack Detection Under Web-Based LLM Workload via L1D Miss Pending Event

**DOI:** 10.3390/e28030254

**Published:** 2026-02-26

**Authors:** Jiajia Jiao, Quan Zhou, Yulian Li

**Affiliations:** College of Information Engineering, Shanghai Maritime University, No. 1550 Haigang Avenue, Shanghai 201306, China; 202430310242@stu.shmtu.edu.cn (Q.Z.); ylli@shmtu.edu.cn (Y.L.)

**Keywords:** Spectre attack, attack detection, web-based LLMs, HPC events

## Abstract

As Large Language Models (LLMs) become increasingly integrated into web environments, they introduce complex microarchitectural noise that challenges existing hardware security mechanisms. This paper investigates the impact of concurrent web-based LLM workloads on the detection accuracy of Spectre attacks. Firstly, we constructed a representative dataset by executing multiple web-accessible LLMs (e.g., DeepSeek, Kimi, Doubao and Qwen) alongside Spectre attacks, capturing the specific interference patterns introduced by these AI workloads. Experimental analysis reveals that traditional Hardware Performance Counter (HPC)-based detectors, relying primarily on branch prediction and Last-Level Cache (LLC) events, suffer significant accuracy degradation due to the masking effects of LLM-induced noise. To address this limitation, we then propose a novel Spectre attack detector Spec-LAMP via augmenting conventional HPC feature sets with the L1D Miss Pending event. This new metric specifically captures unresolved speculative memory dependencies, a distinctive characteristic of Spectre attacks that remains discernible even under web-accessible LLM interference. Comparative statistical analysis demonstrates that incorporating this event significantly enhances the separability between malicious and benign executions. Finally, experimental results show that our proposed feature augmentation effectively restores detection performance, increasing average accuracy from 85.15% to 98.43% and demonstrating superior robustness compared to traditional approaches in realistic web-based LLM scenarios.

## 1. Introduction

In recent years, web-based Large Language Models (LLMs) [1] have rapidly transitioned from experimental systems to widely deployed, user-facing services. Platforms such as DeepSeek [2], Kimi [3], Doubao [4], and Qwen [5] are now routinely accessed through web browsers, providing real-time natural language interaction to millions of users. However, the pervasive deployment of web-based LLMs fundamentally complicates the detection of Spectre attacks. The execution characteristics of web-based LLMs, such as deep memory hierarchies, highly dynamic control flow, and extensive speculative execution, introduce substantial microarchitectural noise. This noise directly affects Hardware Performance Counter (HPC) events [6], which are commonly used as indicators in existing Spectre detection mechanisms. As a result, the reliability of traditional HPC-based Spectre detectors is significantly undermined in realistic web-based LLM execution environments.

Spectre attacks [7] are a class of speculative execution vulnerabilities that exploit transient execution to leak sensitive information through microarchitectural side channels. To detect such attacks at runtime, prior research has extensively explored approaches based on HPCs. These methods typically monitor indicators such as branch miss rates and Last-Level Cache (LLC) miss rates [8,9,10], and have demonstrated high detection accuracy in relatively controlled or low-noise environments. However, this assumption no longer holds in the presence of concurrent web-based LLM workloads. Our experimental results show that LLM-induced execution patterns significantly distort the distributions of commonly used HPC events. In particular, the intensive control-flow speculation and memory access behavior of LLM inference workloads can closely resemble the microarchitectural footprints of Spectre attacks, thereby masking malicious signals. As a consequence, detectors relying primarily on branch prediction and LLC-related events experience a substantial degradation (detection accuracy decreasing from 99.84% to 85.15%) in accuracy under realistic web-accessible LLM interference.

In this paper, we propose Spec-LAMP, a novel Spectre attack detection approach that augments conventional HPC feature sets with the L1 Data Cache Miss Pending (L1D Miss Pending) event. This event captures the number of outstanding L1 data cache misses whose memory dependencies have not yet been resolved. Crucially, such unresolved speculative memory accesses are a defining characteristic of Spectre attacks, which deliberately trigger transient loads to unauthorized memory locations and rely on delayed resolution to leak information via side channels.

To evaluate our approach, we construct a representative dataset by executing various popular web-based LLMs with Spectre attacks concurrently, capturing realistic microarchitectural interference patterns. Experimental results demonstrate that traditional HPC-based detectors suffer notable accuracy degradation in this setting, whereas Spec-LAMP achieves robust performance. Specifically, our feature augmentation increases average detection accuracy from 85.15% to 98.43%, significantly outperforming conventional methods under web-based LLM workloads.

Our main contributions are summarized as follows:

- **A comprehensive dataset** is constructed by combining multiple web-based LLM workloads with a wide range of Spectre attacks, enabling systematic analysis under realistic microarchitectural interference.
- **The interaction between Spectre attack detection and concurrent web-based LLM execution** is systematically investigated, providing new insights and research directions for attack detection in AI-intensive environments.
- **A detailed statistical analysis of L1, L2, and L3 cache related HPC events** is conducted to identify the most sensitive and robust indicators of Spectre attack behavior under LLM-induced noise.
- **A novel Spectre attack detection method Spec-LAMP** is proposed, significantly improving detection accuracy in the presence of web-based LLM workloads. The source code of Spec-LAMP is publicly available at: https://github.com/Hamster-K/Spec-LAMP (accessed on 17 January 2026).

The remainder of this paper is organized as follows. Section 2 introduces the background of web-based LLM workloads, Spectre attacks and their variants, and the use of HPC-based detection. Section 3 presents the motivation, feature ranking and selection in Spec-LAMP, and the overall Spec-LAMP detection framework in details. Section 4 describes the experimental setup and evaluates the detection performance and robustness of Spec-LAMP under different web-based LLM interferences. Section 5 reviews related work on HPC-based Spectre detection. Finally, Section 6 concludes the paper and discusses directions for future research.

## 2. Background

### 2.1. Web-Based LLMs

Web-based LLMs refer to LLM services that are accessed through web browsers or web interfaces and invoked via network-based application programming interfaces (APIs). Representative examples include widely deployed services such as DeepSeek [2], Kimi [3], Doubao [4], and Qwen [5], which provide interactive LLM capabilities through browser-based front ends. In such settings, model inference is performed on remote servers, while client-side execution primarily involves request construction, data serialization, network communication, and response processing within the browser’s runtime environments. These operations are orchestrated through modern web technologies, including JavaScript engines and browser runtime systems, and are tightly interleaved with user interaction and system-level resource management.

From a microarchitectural perspective, although the core neural network inference is offloaded to remote servers, web-based LLM interactions still exhibit distinctive execution characteristics on the client side. The frequent invocation of LLM APIs triggers intensive browser activity, including dynamic JavaScript execution, asynchronous event handling, just-in-time (JIT) compilation, and memory allocation for network buffers and response parsing. Such activities introduce irregular control flow, frequent data-dependent branching, and bursty memory access patterns.

When executed alongside other workloads, these browser-induced behaviors place sustained pressure on branch predictors, translation lookaside buffers (TLBs), and multi-level cache hierarchies [11]. As a result, the widespread deployment of web-based LLMs introduces a substantial source of microarchitectural noise. This noise manifests as elevated branch misprediction rates, increased cache miss events, and a large number of unresolved memory accesses that coexist with benign web activity [12]. While these effects do not compromise functional correctness, they fundamentally alter the microarchitectural footprint of normal execution.

This shift in the execution environment has important security implications. Many existing runtime Spectre detection mechanisms rely on HPCs to identify anomalous speculative behaviors. However, the microarchitectural interference induced by concurrent web-based LLM workloads can obscure or mask the distinctive signatures of Spectre attacks, thereby degrading detection reliability [13]. Consequently, understanding and characterizing the microarchitectural impact of web-based LLMs is essential for designing robust Spectre detection mechanisms in realistic deployment scenarios.

### 2.2. Spectre Attacks and Variants

Spectre attacks [7,14] are a class of transient execution attacks that exploit speculative execution mechanisms in modern processors to leak sensitive information across security boundaries. Modern CPUs aggressively speculate on control-flow and memory access decisions to improve performance. Although incorrect speculative execution is eventually squashed at the architectural level, its microarchitectural side effects, such as cache state modifications and branch predictor updates, may persist and be observed through side channels [15].

Since the initial disclosure, several major Spectre variants have been identified, each exploiting different speculative mechanisms and prediction structures.

- **Spectre-PHT (Variant 1)**: This variant exploits the processor’s branch predictor, specifically the Pattern History Table (PHT), to mispredict conditional branches [7]. Attackers train the predictor to speculatively execute code that accesses secret data, which is then leaked through cache side channels.
- **Spectre-BTB (Variant 2)**: This variant targets indirect branches by poisoning the Branch Target Buffer (BTB) [7,16]. By redirecting the speculative control flow to a gadget containing secret-dependent memory accesses, attackers can leak information based on the outcomes of this speculative execution.
- **Meltdown (Variant 3)**: Meltdown, exploits delayed permission checks on speculative memory loads [17]. During speculative execution, unauthorized memory accesses may transiently succeed and populate cache lines before permission checks are enforced. Although Meltdown is sometimes categorized separately from Spectre, it shares the same fundamental principle of transient execution leakage.
- **Spectre-SSB (Variant 4)**: This variant abuses memory disambiguation in speculative execution [16]. It allows a load instruction to speculatively bypass a previous store, potentially exposing stale or sensitive values that should have been overwritten, thereby leaking information across security boundaries.

### 2.3. HPC-Based Detection for Spectre Attack and Variants

HPCs [6] provide fine-grained visibility into microarchitectural activities of modern processors, such as instruction execution, branch behavior, and cache utilization. Originally designed for performance analysis and optimization, HPCs have been widely adopted for security monitoring due to their low overhead and ability to capture subtle microarchitectural side effects induced by malicious execution. Specifically for Spectre attacks, HPCs offer a unique vantage point to observe the transient microarchitectural changes that accompany speculative execution vulnerabilities. By continuously monitoring selected hardware events at runtime, security mechanisms can detect anomalous execution patterns associated with microarchitectural attacks without requiring source code instrumentation or program modification.

Spectre attacks, regardless of their specific variant fundamentally rely on triggering incorrect speculative execution to manipulate microarchitectural state (like branch predictors or caches) and then observing these changes via side channels. This transient execution leaves distinct footprints in HPC metrics. All Spectre variants typically involve loads from unauthorized memory regions that transiently succeed, affecting cache hierarchies or memory disambiguation units, which can be observed through corresponding cache miss/hit events or pending memory accesses. Therefore, by analyzing patterns in HPCs such as branch misprediction rates, and cache miss rates detectors can infer the presence of a Spectre attack.

**Figure 1:**
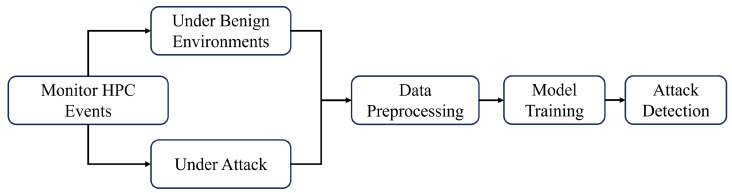
Overview of the HPC-based Detection Framework for Spectre Attack and Variants.

As shown in Figure 1, the HPC-based detection first monitors HPC events under both benign and attack environments. The collected data is subsequently preprocessed and fed into a learning model for training. The resulting detector is then able to effectively distinguish malicious attack behavior from normal execution.

Performance monitoring tools, leveraging HPCs, typically support two primary monitoring modes: sampling mode and counting mode [18]. Sampling mode periodically records hardware events along with program context, enabling fine-grained analysis but often incurring higher overhead. In contrast, counting mode aggregates the total number of selected hardware events over a fixed time window, providing a lightweight and low-overhead view of global microarchitectural activity.

This work focuses on counting-based HPC monitoring, which is widely adopted in security monitoring due to its efficiency and suitability for continuous observation. By collecting global event counts within fixed intervals, counting mode captures the statistical characteristics of speculative execution attacks while remaining practical for real-world deployment.

## 3. Proposed Methodology

### 3.1. Motivation

Existing Spectre detection approaches [8,10,19] primarily leverage HPC features, such as branch misprediction rates and LLC miss rates, to distinguish speculative attacks from benign executions. Under relatively controlled runtime environments, these microarchitectural indicators exhibit clear and stable statistical patterns, thereby enabling effective separation between attack and non-attack behaviors.

**Figure 2:**
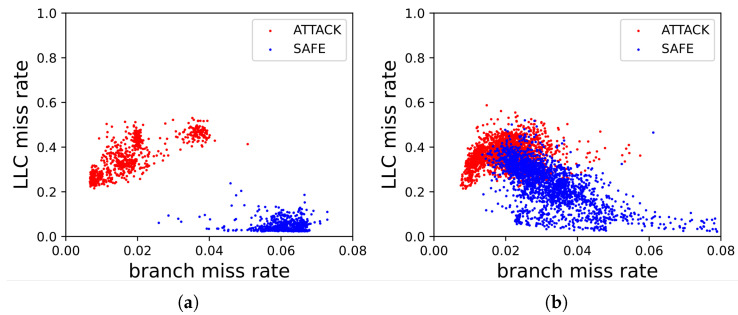
Impact of Web-based LLM Noise on Traditional Spectre Detection Features. (**a**) Pure Environment. (**b**) Web-based LLM as Noise.

Figure 2 illustrates this phenomenon by contrasting two runtime scenarios. In a pure execution environment without significant background interference, the distributions of attack and benign samples in the feature space formed by branch miss rate and LLC miss rate are well separated. As shown in Figure 2a, attack executions cluster in a distinct region characterized by elevated LLC miss rates and lower branch miss rates, while benign executions form a separate cluster. This clear separability allows conventional classifiers to construct reliable decision boundaries and achieve high detection accuracy.

However, this assumption no longer holds in the presence of web-based LLM workloads. When web-accessible LLM services are executed concurrently and act as background noise, the resulting microarchitectural interference substantially perturbs traditional HPC features. As shown in Figure 2b, both attack and benign executions exhibit significantly more concentrated and overlapping distributions. The intensive memory accesses, complex control flow, and speculative execution activities induced by web-based LLM workloads distort branch prediction and cache behavior, effectively masking the distinctive microarchitectural signatures of Spectre attacks. As a consequence, the overlap between positive (attack) and negative (benign) classes increases dramatically, making reliable detection difficult for traditional HPC-based methods. Even when trained with labeled data, detectors relying solely on branch and LLC events struggle to generalize under such noisy conditions, with detection accuracy decreasing from 99.84% to 85.15%. This observation highlights a fundamental limitation of existing detection techniques when deployed in realistic web-based LLM execution environments.

These findings motivate the need for more robust detection features that remain discriminative under intense microarchitectural noise. In particular, it becomes essential to identify hardware events that can directly capture unresolved speculative memory dependencies, which are intrinsic to Spectre attacks and less susceptible to interference from web-based LLM workloads.

### 3.2. Hybrid Statistical HPC Feature Ranking and Selection for Spec-LAMP

To overcome the limitations of traditional Spectre detection in noisy web-based LLM environments, our proposed method, Spec-LAMP, introduces a systematic approach to identify and rank highly discriminative HPC features. Traditional metrics, such as branch prediction and LLC miss rates, are highly susceptible to interference from the intensive and heterogeneous execution patterns of LLM workloads, which distort cache and branch predictor behavior. This leads to significant overlap between attack and benign execution profiles. Consequently, identifying HPC events that remain stable and discriminative under such web-based LLM environments becomes essential for reliable Spectre detection.

To address this challenge, our analysis focuses on memory- and speculation-related HPC events at the L1 and L2 cache levels. As summarized in Table 1, a comprehensive set of cache-related events is selected from the Intel Software Developer’s Manual [20], covering the L1 data cache L1D, the L2 cache, and the LLC. Given that LLC-related events have been extensively explored in prior HPC-based Spectre detection studies, the analysis primarily focuses on events associated with the L1 and L2 cache levels. These lower-level cache events are more closely coupled with speculative memory access behavior and are therefore more likely to capture intrinsic characteristics of Spectre attacks while being less affected by shared resource contention, introduced by web-based LLM workloads.Our selection process begins with a qualitative analysis to define a candidate set, followed by a rigorous quantitative statistical framework to pinpoint the single most discriminative and robust HPC feature for our detection model.

**(1) An initial qualitative analysis for a small HPC set.** Based on the above considerations, the analysis focuses on a representative set of L1 and L2 cache events, including L1D MISS PENDING, L1D REPLACEMENT, L2 RQSTS MISS, L2 RQSTS ALL DEMAND MISS, L2 RQSTS REFERENCES, and L2 RQSTS ALL DEMAND REFERENCES. These events are selected to comprehensively capture different aspects of memory access behavior and cache hierarchy interactions that are closely related to speculative execution.

Specifically, L1D MISS PENDING reflects the number of outstanding L1 data cache misses that remain unresolved during execution, directly indicating speculative load activity and unresolved memory dependencies inherent to Spectre attacks. L1D REPLACEMENT captures cache line eviction behavior at the L1 data cache level, providing insight into cache pressure and data locality effects induced by speculative memory accesses. At the L2 cache level, L2 RQSTS MISS and L2 RQSTS ALL DEMAND MISS quantify miss behavior for both general and demand-driven requests, while L2 RQSTS REFERENCES and L2 RQSTS ALL DEMAND REFERENCES characterize overall access intensity and memory traffic toward lower cache levels.

**(2) A rigorous quantitative statistical analysis for selecting robust HPC feature.** Hardware security literature [21,22,23] consistently underscores the significance of principled feature selection for enhancing classifier performance in attack classification tasks. To identify the most sensitive and discriminative event among the selected L1 and L2 cache metrics, a statistical evaluation framework is employed to quantify the distributional differences between attack and benign executions under concurrent web-based LLM workloads. For each candidate HPC event, multiple complementary statistical indicators are computed, including the t-statistic, *p*-value, Kolmogorov–Smirnov (KS) statistic, and the corresponding KS *p*-value.

Specifically, the t-statistic is used to measure the difference in mean values between the attack and benign samples, defined as:

(1) $$t=\frac{\mu_{\mathrm{attack}}-\mu_{\mathrm{benign}}}{\sqrt{\frac{\sigma_{\mathrm{attack}}^{2}}{n_{\mathrm{attack}}}+\frac{\sigma_{\mathrm{benign}}^{2}}{n_{\mathrm{benign}}}}}$$

where μ, $\sigma^{2}$, and *n* denote the sample mean, variance, and number of observations, respectively. The associated *p*-value indicates the statistical significance of the observed difference under the null hypothesis that the two distributions share the same mean. Formally, the *p*-value is defined as:

(2) $$p_{t}=\mathrm{Pr}\left(\left|T|\geq \right|t_{\mathrm{obs}}||H_{0}\right),$$

where *T* follows a t-distribution with degrees of freedom determined by the sample sizes and variances, and $t_{\mathrm{obs}}$ denotes the observed t-statistic.

In addition to mean-based comparison, the KS test is adopted to assess the overall distributional divergence between attack and benign executions. The KS statistic is defined as:

(3) $$D_{\mathrm{KS}}=\underset{x}{\sup}\left|F_{\mathrm{attack}}\left(x\right)-F_{\mathrm{benign}}\left(x\right)\right|$$

where $F_{\mathrm{attack}}\left(x\right)$ and $F_{\mathrm{benign}}\left(x\right)$ represent the empirical cumulative distribution functions of the corresponding samples. The KS *p*-value evaluates the likelihood that both samples are drawn from the same underlying distribution, and is defined as:

(4) $$p_{\mathrm{KS}}=\mathrm{Pr}\left(D\geq D_{\mathrm{KS}}|H_{0}\right),$$

where *D* follows the Kolmogorov distribution under $H_{0}$.

By jointly considering the magnitude of the t-statistic, the statistical significance indicated by $p_{t}$, and the distributional separability captured by the KS statistic and $p_{\mathrm{KS}}$, the sensitivity and robustness of each HPC event can be systematically assessed. Events that exhibit consistently large test statistics and statistically significant *p*-values are regarded as more reliable indicators for Spectre detection under web-based LLM interference.

Algorithm 1 presents a systematic statistical framework to rank and select robust HPC events for Spectre detection under web-based LLM workloads. The algorithm takes a set of candidate HPC events and execution traces collected under attack and benign conditions as input.
**Algorithm 1: **Statistical Ranking of HPC Events under Web-based LLM Workloads
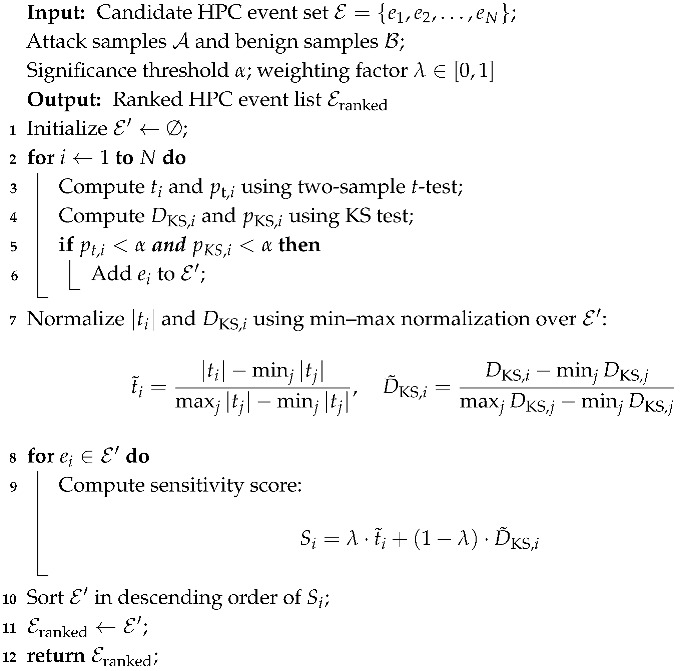


The procedure begins by initializing an empty set of refined candidate features $E^{\prime }$. From lines 2 to 6, for each candidate event, we conduct a two-sample *t*-test and a KS test to evaluate its ability to distinguish between attack and benign executions. This dual-test approach captures both mean-level behavioral deviations and non-Gaussian distributional shifts. HPC features that satisfy the statistical significance threshold of α set to 0.05 are added to the target set $E^{\prime }$. Such a significance threshold represents a widely accepted balance between sensitivity and confidence in microarchitectural and systems security studies, ensuring that only events exhibiting consistent and reliable separability are retained.

In line 7, we then apply min–max normalization to the absolute t-statistics and KS statistics across all HPC features in $E^{\prime }$ to eliminate scale disparities. Subsequently, as described in lines 8 and 9, we compute a composite sensitivity score by linearly combining these normalized metrics. The weighting factor λ set to 0.5, with equal importance to mean-level separation and distributional divergence, providing a balanced characterization of feature discriminability. Finally, the events in $E^{\prime }$ are ranked in descending order based on their sensitivity scores, and the sorted list is returned to facilitate the selection of the most discriminative and robust HPC features for Spectre detection in noisy web-based LLM environments.

### 3.3. Overall Spec-LAMP Framework

**Figure 3:**
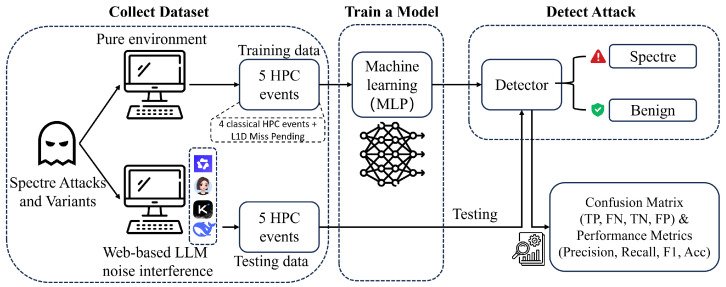
Overview of the Proposed Spec-LAMP Framework for Spectre Detection under Web-based LLM Interference.

With the new robust HPC feature L1D MISS PENDING identified in Spec-LAMP, we can complete the overall framework involving the three steps: data collection, model training and attack detection in Figure 3.

#### 3.3.1. Dataset Collection

Spec-LAMP collects microarchitectural behavior from two environments: a pure environment for model training and a noisy, web-based LLM-interfered environment for evaluating detection performance. In both settings, Spectre attacks and benign executions are repeatedly executed to ensure comprehensive behavioral coverage.

In the pure environment, we capture baseline microarchitectural characteristics of Spectre attacks and benign programs without external interference. To emulate realistic deployment scenarios and assess robustness, we concurrently introduce web-based LLM workloads as background noise in the noisy environment. These workloads intensely perturb microarchitectural states, significantly complicating attack detection.

Across both environments, a fixed set of five sensitive HPC events previously identified through rigorous statistical analysis, are continuously monitored and recorded. This set includes four classical HPC events [8] (branches, branch misses, LLC references, and LLC misses) and our newly proposed L1D miss pending event, which captures speculative memory access pressure at the L1 data cache level. HPC traces from the pure environment exclusively form the training data, while those from the noisy, LLM-interfered environment serve as testing data. This clear training–testing separation, leveraging data from diverse and potentially unseen LLM workloads, directly evaluates the robustness of Spec-LAMP against real-world interference. This approach underscores our method’s strong generalizability, enabling effective Spectre detection even under unseen web-based LLM workloads.

#### 3.3.2. Model Training with Selected HPC Events

In the training stage, HPC measurements collected from the pure environment are utilized to train a machine learning model. The objective of this model is to learn discriminative patterns differentiating Specter attacks from benign executions. Each data sample is represented as a feature vector comprising both raw five HPC events and two derived features: branch miss rate and LLC miss rate. Specifically, the branch miss rate is computed from recorded branches and branch misses, while the LLC miss rate is derived from LLC references and LLC misses. These features collectively capture the fine-grained cache and memory-level behaviors induced by speculative execution.

Spec-LAMP adopts a multilayer perceptron (MLP) as the underlying classifier. MLPs are well suited for modeling nonlinear relationships among multiple HPC features, which commonly arise from the complex interactions between speculative execution, cache hierarchies, and branch prediction mechanisms. Prior studies on HPC-based Spectre detection [9,24,25] have shown that MLP-based models consistently achieve strong detection performance and robustness compared with linear classifiers and shallow statistical models, making them a practical and effective choice for microarchitectural anomaly detection.

For this training, we employ a simple MLP to establish a baseline for evaluating feature effectiveness. The architecture consists of two fully connected hidden layers with 12 and 8 neurons, respectively, each followed by a ReLU activation function. The output layer features a single neuron with a sigmoid activation for binary classification. The model is trained for 10 epochs using the Adam optimizer and binary cross-entropy loss, following a fixed training protocol. During this training phase, the dataset is randomly split into 70% for training and 30% for validation (testing this specific model’s learning progress), ensuring no overlap between the two sets. The network’s depth and width are intentionally kept minimal to mitigate overfitting and ensure that detection performance primarily reflects the intrinsic quality of the selected features rather than excessive model capacity.

By training the model exclusively on clean data from the pure environment, Spec-LAMP establishes a stable decision boundary that captures intrinsic Spectre-induced microarchitectural anomalies, rather than artifacts specific to noisy environments. This strategic design choice significantly enhances the model’s generalization capability when deployed in complex, noisy real-world web-based LLM execution environments.

#### 3.3.3. Attack Detection and Evaluation

In the detection stage, the pre-trained model is deployed to evaluate testing samples collected specifically under web-based LLM interference. Leveraging the robust microarchitectural patterns learned from clean data, the detector classifies each incoming sample as either a Spectre attack or a benign execution. This step is critical for verifying whether the discriminative features identified in the pure environment remain effective when subjected to intense background noise.

To quantitatively evaluate detection performance, Spec-LAMP reports a confusion matrix, including true positives (TPs), false positives (FPs), true negatives (TNs), and false negatives (FNs). Standard performance metrics, including Precision, Recall, F1-score, and Accuracy, are further derived to provide a comprehensive assessment of detection effectiveness under noisy conditions.

Overall, Spec-LAMP integrates statistically guided feature selection, learning-based modeling, and noise-aware evaluation into a unified framework. By explicitly considering web-based LLM interference, the proposed approach advances practical and robust Spectre detection in modern, complex web-driven computing environments.

## 4. Results and Analysis

### 4.1. Experimental Setup and Dataset Construction

All experiments were conducted on a dedicated server platform, with hardware, and software configurations are summarized in Table 2. The system is equipped with an Intel Xeon Silver 4210 (Cascade Lake) processor, featuring 20 cores at 2.2 GHz, and 125.5 GiB of main memory, providing sufficient resources to support long-running Spectre attack executions and concurrent web-based workloads. The operating system is Ubuntu 18.04.6 LTS (64-bit) with Linux kernel 5.4.0-146, and hardware performance counter data are collected using perf 5.4.229. This setup enables stable and fine-grained monitoring of microarchitectural events, including cache- and speculation-related HPCs that are critical for Spectre detection. All data processing, feature extraction, and model training are implemented in Python 3.10, with experiments managed using PyCharm Professional 2022.1.3. Overall, this server-class environment reflects realistic deployment conditions, ensuring that the experimental results are both representative and reproducible.

To evaluate the robustness of Spec-LAMP under realistic web-based interference, we construct a comprehensive dataset by collecting HPC traces under both pure execution environments and web-based LLM–interfered environments, with detailed experimental configurations summarized in Table 2.

We consider four representative Spectre variants: Spectre-PHT, Spectre-BTB, Meltdown, and Spectre-SSB, each executed alongside corresponding benign workloads. To model realistic background noise, four widely used web-based LLM services (DeepSeek, Kimi, Doubao, and Qwen) are accessed through a browser and run concurrently with the target programs. These interactions generate representative browser-side workloads, including JavaScript execution, network communication, and runtime resource management, closely reflecting practical deployment scenarios in which security monitoring must coexist with web-based AI services.

All LLM services are accessed via Google Chrome with default settings, and interactions are fully automated using Selenium to ensure repeatability and consistency. The same predefined set of comprehension- and reasoning-oriented questions is used across all services. To emulate realistic user behavior, the automation framework dynamically detects response completion and introduces a short idle interval between consecutive queries. Non-essential applications, background processes, browser extensions, and additional tabs are disabled to minimize confounding interference, ensuring that the observed microarchitectural noise primarily originates from LLM interactions.

For each Spectre variant, HPC traces are collected under two conditions: (1) a pure environment, where only the attack or benign program is executed, and (2) a web-based LLM interference environment, where one LLM service runs concurrently. Each execution instance is profiled using a fixed set of selected HPC events, including four conventional Spectre-related events and the newly proposed L1D miss pending event, along with two derived rate-based features. All traces are labeled as attack or benign accordingly.

HPCs are collected using perf in counting mode with a fixed readout interval of 500 ms. Each labeled sample corresponds to a single, independent execution instance, obtained from a fresh invocation of the target program rather than from slicing long-running traces. This design preserves sample independence and avoids artificial sample inflation.

In terms of dataset scale, for each Spectre variant, we collect traces under one pure environment and four LLM-interfered environments, with balanced numbers of attack and benign samples per condition. This results in approximately 6000 samples per variant and about 24,000 labeled samples in total across all variants.

Following the design of Spec-LAMP, traces from the pure environment are used exclusively for training, while traces collected under web-based LLM interference are reserved for testing. This strict environment-level separation enables a rigorous evaluation of generalization performance and robustness under complex, noise-intensive conditions.

### 4.2. Spec-LAMP Detection Performance Under Spectre Variants and Web-LLM Interference

Table 3 summarizes the detection accuracy of the baseline method and the proposed Spec-LAMP across different Spectre variants and Web-based LLM interference environments.

**Overall Detection Performance.** Across all evaluated scenarios, Spec-LAMP consistently outperforms the baseline detector. In terms of average accuracy over all Spectre variants and execution environments, the baseline achieves 85.15%, whereas Spec-LAMP improves the performance to 98.5%, yielding an accuracy gain of more than 13 percentage points. This substantial improvement indicates that Spec-LAMP is significantly more resilient to the execution noise introduced by concurrent Web-based LLM workloads.

**Impact of Web-LLM Interference.** In the absence of Web-LLM interference, both methods achieve near-perfect accuracy (99.84% for the baseline and 99.98% for Spec-LAMP), confirming that Spectre detection is relatively straightforward under clean execution conditions. However, once Web-LLM workloads are introduced, the baseline detector suffers a pronounced performance degradation.

Under Web-LLM environments such as DeepSeek, Kimi, Doubao, and Qwen, the baseline accuracy drops significantly, reaching as low as 75.89% under Qwen and 79.15% under Doubao. This behavior suggests that traditional branch- and cache-based features are highly sensitive to microarchitectural noise induced by complex browser-side LLM execution.

In contrast, Spec-LAMP maintains consistently high detection accuracy across all Web-LLM settings, with average accuracies above 96.3%, even under the most challenging Qwen environment. These results demonstrate the strong robustness and generalization capability of Spec-LAMP in noisy, realistic execution environments.

**Impact of Spectre Variants.** From a per-attack perspective, the baseline method exhibits uneven detection capability across different Spectre variants. While Spectre-PHT and Spectre-BTB remain detectable with moderate accuracy in some environments, the detection performance for Spectre-SSB degrades substantially under Web-LLM interference.

By contrast, Spec-LAMP achieves near-perfect detection accuracy across all four attack variants. Notably, Spectre-SSB and Meltdown consistently reach 100% accuracy in most Web-LLM environments, indicating that Spec-LAMP captures stable and discriminative microarchitectural signatures that are less affected by speculative execution noise and background interference.

These results highlight that relying solely on traditional branch- and cache-related metrics is insufficient in noisy Web-based execution environments. By incorporating L1D miss pending behavior, Spec-LAMP effectively separates speculative attack patterns from background Web-LLM activity, resulting in superior robustness and generalization. Overall, Spec-LAMP significantly outperforms the baseline detector under realistic Web-LLM workloads, making it a practical and reliable solution for speculative attack detection in modern web-centric systems.

### 4.3. Spec-LAMP Robustness Under Web-LLM Interference

#### 4.3.1. Impact of LLM Variations Within the Same Platform

In the previous section, we showed that different web-based LLM services introduce varying degrees of interference to Spectre detection. To further isolate the root cause of this behavior, we examine whether the observed performance variations stem from differences between LLM models or are primarily determined by the web-based execution platforms themselves.

For each web-based LLM service, we select two representative models for evaluation. Specifically, DeepSeek-Chat and DeepSeek-Reasoner are used for DeepSeek, Kimi-K1.5 and Kimi-K2 for Kimi, Doubao-seed and Doubao-seedThinking for Doubao, and Qwen-Max and Qwen-Flash for Qwen. Each model is continuously executed through browser-based interfaces alongside attack or benign programs, and detection performance is evaluated using accuracy, precision, recall, and F1-score.

**Figure 4:**
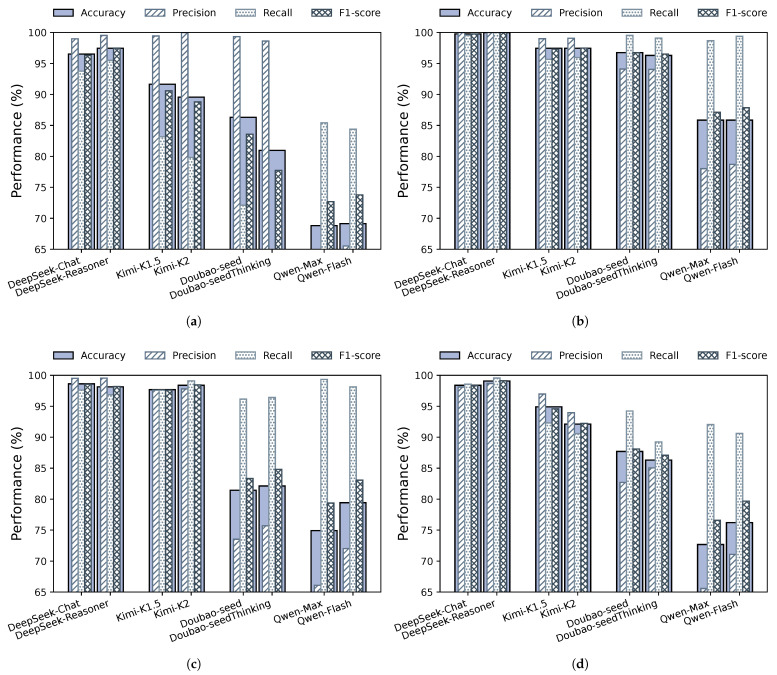
Robustness of Spec-LAMP under different web-based LLM workloads across four Spectre variants. (**a**) Spectre-PHT detection under different Web-LLM workloads. (**b**) Spectre-BTB detection under different Web-LLM workloads. (**c**) Meltdown detection under different Web-LLM workloads. (**d**) Spectre-SSB detection under different Web-LLM workloads.

Figure 4 illustrates the detection results across different LLMs for four representative speculative execution attacks, namely Spectre-PHT, Spectre-BTB, Meltdown, and Spectre-SSB. As illustrated, models belonging to the same web-based service yield highly consistent detection performance across all evaluation metrics. The variations in accuracy, precision, recall, and F1-score are minimal, indicating stable detector behavior under model-level changes.

These results indicate that the impact of web-based LLM interference on HPC-based Spectre detection is dominated by the characteristics of the web execution environment, rather than by the specific LLM deployed. This observation aligns with prior work by Gulmezoglu et al. [12], which shows that browser-based workloads exhibit distinctive and stable microarchitectural footprints primarily arising from JavaScript execution, runtime scheduling, memory management, and browser-controlled resource activities, rather than application-level semantics.

Consequently, the dominant source of microarchitectural noise captured by HPCs in our experiments originates from the browser execution environment itself, explaining why different models within the same web-based LLM service produce similar interference patterns. Overall, these results demonstrate that Spec-LAMP maintains robust detection performance across different LLMs within the same platform, while the degree of performance degradation varies across platforms. This further confirms that web-based LLM interference is platform-dependent, and that Spec-LAMP can effectively generalize across heterogeneous and realistic web execution environments.

#### 4.3.2. Cross-Service Generalization Under Leave-One-Service-Out (LOSO) Evaluation

To further assess the robustness and cross-service generalization capability of Spec-LAMP, we conduct an additional evaluation using a leave-one-service-out (LOSO) testing strategy. In this setting, the classifier is trained on traces collected under Web-based LLM interference from three services and evaluated on a fourth, previously unseen service. This design explicitly examines whether the proposed detection approach can generalize across heterogeneous web-based execution environments characterized by distinct interference patterns.

Specifically, each Web-based LLM service is held out in turn as the test domain, while traces from the remaining three services are used for training. The held-out service introduces browser-level execution behaviors, scheduling patterns, and memory access characteristics that are not observed during training, thereby constituting a strict and realistic cross-service generalization test.

Table 4 summarizes the LOSO evaluation results across four representative speculative execution attacks, including Spectre-PHT, Spectre-BTB, Meltdown, and Spectre-SSB. The LLM listed in the first column denotes the web-based LLM service that is held out and used exclusively as the test domain in each LOSO setting. As shown in the table, Spec-LAMP consistently achieves high detection accuracy across all held-out services, with per-service average accuracies ranging from 95.14% to 98.73%, and an overall average accuracy of 96.89%.

Notably, even without exposure to traces from the target service during training, Spec-LAMP maintains strong detection performance across all attack variants. The consistently high accuracy indicates that the learned detection model does not overfit to service-specific noise patterns. Instead, it captures invariant microarchitectural behaviors intrinsic to speculative execution attacks.

These results demonstrate that Spec-LAMP effectively captures invariant microarchitectural behaviors associated with speculative execution attacks, enabling reliable detection under previously unseen Web-based LLM interference. The LOSO evaluation therefore provides strong evidence of Spec-LAMP’s robustness and cross-service generalization capability in realistic and heterogeneous web execution environments.

### 4.4. HPC Feature Importance Analysis of Spec-LAMP

**Overall Feature Ranking.** Table 5 summarizes the overall feature ranking under Web-based LLM interference. A clear separation emerges at the top of the list: *L1D miss pending* ranks first with the largest |t| (83.31) and the highest KS statistic (0.87), yielding a normalized score of 1.0. The next-best feature, *LLC miss rate*, while still strong (Rank 2), shows noticeably weaker separation (|t| = 56.97 and KS = 0.648). This gap indicates that *L1D miss pending* provides not only a larger average shift but also a more consistent distribution-level distinction between attack and benign executions under Web-LLM noise. In contrast, deeper-cache indicators become progressively less stable: for example, *L2 miss rate* and *L2 demand miss rate* fall to the bottom of the ranking with much smaller KS values (0.142 and 0.125), suggesting that their distributions are more easily blurred by browser-induced activity.

The superior performance of *L1D miss pending* can be attributed to its close coupling with speculative execution behavior. Spectre attacks intentionally prolong speculative execution windows, generating unresolved L1 data cache misses that accumulate as pending miss entries. This microarchitectural effect manifests before cache line resolution, eviction, or replacement, making it inherently less sensitive to background activities such as JavaScript execution, memory allocation, and browser runtime scheduling. As a result, L1D miss pending captures a more intrinsic and speculation-aware manifestation of Spectre-induced anomalies than miss rate based metrics derived from deeper cache levels.

It is noted that although *p*-values are not used as ranking scores, they play a critical role in ensuring the statistical validity of the feature selection process. For each HPC event, we compute the *p*-value from Welch’s two-sample *t*-test ($p_{t}$) and from the KS test ($p_{ks}$), and an event is retained only when both satisfy the joint significance threshold λ=0.05. In Table 5, all candidate events pass this criterion, implying that the observed separations are statistically reliable on our pooled dataset. Therefore, Table 5 should be interpreted as comparing how strongly different statistically valid events separate the two classes, rather than merely whether they differ.

**Figure 5:**
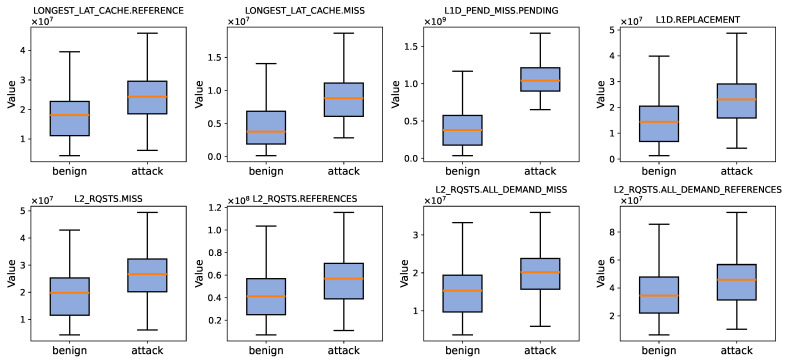
Distributional Separation of Candidate HPC Events between Attack and Benign Executions under Web-based LLM Interference.

**Distributional Validation of the Ranking.** Figure 5 visually corroborates these ranking results. The box plots show that *L1D miss pending* exhibits the most pronounced shift between attack and benign executions with limited overlap, matching its dominant KS statistic (0.87) in Table 5. Meanwhile, events with lower KS values show visibly larger overlaps, consistent with their reduced discriminability in the ranking. Taken together, Figure 5 confirms that the ordering in Table 5 reflects genuine, distribution-level separations rather than artifacts of a single summary statistic.

**End-to-End Impact on Detection Performance.** Table 6 evaluates whether the ranking in Table 5 translates into end-to-end detection improvements when combined with a practical baseline feature set. The baseline model using four classical events (branches, branch misses, LLC references, LLC misses) achieves 85.15% accuracy. When augmenting this baseline with one additional event, only *L1D miss pending* produces a substantial gain, increasing accuracy to 98.43% (13.28% absolute improvement). This improvement is accompanied by simultaneous gains in precision (0.8157 → 0.9752), recall (0.9084 → 0.9939), and F1-score (0.8595 → 0.9845), indicating that the added feature reduces both false positives and false negatives. In contrast, adding other cache-related events yields only marginal improvements (e.g., accuracies around 86–90%), consistent with their lower ranks and weaker KS separation in Table 5.

Overall, the three results are mutually consistent: Table 5 identifies *L1D miss pending* as the most discriminative event (KS = 0.87), Figure 5 confirms this dominance through the clearest distributional separation, and Table 6 demonstrates that this top-ranked event is also the only one that materially boosts detection when added to the classical baseline. These findings directly motivate Spec-LAMP’s final *4+1* feature design: four baseline HPC events plus *L1D miss pending*.

### 4.5. Discussion

**Practical Implications.** Our findings have practical implications for deploying Spectre detection in modern web-centric systems. We observe that detectors based primarily on branch and LLC related events can become less reliable under concurrent web-based LLM activity, underscoring a key gap between controlled evaluations and realistic deployments. In contrast, the strong performance of for L1D miss pending enhanced Spec-LAMP suggests that speculation-aware, low-level indicators can remain robust under browser-induced noise and are well suited for lightweight runtime monitoring. Spec-LAMP uses standard HPC workflow and does not require code instrumentation or system modifications, enabling practical deployment on commodity platforms. Moreover, robustness across multiple web-based LLM services indicates promising generalization to unseen web workloads.

**Limitations.** Despite its effectiveness, Spec-LAMP has limitations. First, it depends on the availability of specific HPC events (e.g., L1D miss pending), which may limit portability across processor vendors and microarchitectures. Second, the current design focuses on Spectre-style speculative execution attacks and does not aim to cover all classes of microarchitectural attacks. Finally, under extremely heavy or highly variable system load, counter behavior and noise may change, potentially affecting detection stability. Extending the approach to broader architectures and threat models, and further characterizing behavior under more diverse load conditions, are left for future work.

## 5. Related Work

**HPC-based Detection of Spectre and Transient Execution Attacks.** A substantial body of prior work has explored the use of HPCs for detecting Spectre and related transient execution attacks. Li et al. [8,25] demonstrated that microarchitectural anomalies, such as abnormal cache miss behavior and branch mispredictions, can be effectively captured using HPCs and leveraged by machine learning classifiers for online detection. Typically, these approaches rely on cache miss events, branch-related counters, or their derived metrics to distinguish malicious speculative execution from benign workloads. While these methods achieve high accuracy under controlled experimental settings, they typically assume relatively clean execution environments with limited background interference.

Beyond Spectre-specific detectors, a broader line of work has investigated the detection and characterization of transient execution and microarchitectural attacks using timing analysis, performance monitoring, or hybrid software–hardware approaches, as exemplified by Wang et al. [26] and Hassan et al. [27]. These studies demonstrate that transient execution leaves observable microarchitectural footprints, but also show that detection effectiveness is closely tied to workload characteristics and execution context. As a result, the robustness of detection mechanisms remains a key challenge when moving from controlled benchmarks to realistic, noisy environments.

**Evasion and Robustness under Diverse Workloads.** To address adversarial behavior, recent work has investigated evasive Spectre attacks that deliberately manipulate execution patterns to evade detection. As shown by Jiao et al. [9] introduced T-Smade, a two-stage detection framework that adapts to different workload characteristics and improves robustness against evasion techniques. These studies highlight the strong dependency between detection performance and background workloads, demonstrating that naive HPC-based detectors may fail under complex or dynamic execution conditions. However, the workloads considered in these studies are primarily conventional applications and do not account for modern browser-based or web-driven execution environments.

Related observations have also been reported in other noisy execution contexts, such as multi-tenant or cloud environments, where unrelated co-running workloads interfere with microarchitectural signals, as shown by Luo et al. [28] and Volos et al. [29], who analyze the security and isolation challenges arising from shared microarchitectural resources in cloud CPUs. Prior work shows that cache and branch indicators, especially miss rate based metrics derived from deeper cache levels, are particularly susceptible to such interference. However, the workloads considered in these studies are primarily conventional native applications and do not account for modern browser-based or web-driven execution models.

**Impact of Web-based Workloads and Browser Noise.** With the rapid growth of web-based applications, recent studies have begun to examine their impact on microarchitectural attack detection. Jiao et al. [13] systematically evaluated how browser-based LLM services introduce substantial noise into HPC traces, significantly degrading the effectiveness of existing Spectre detectors. This work demonstrates that Web-based LLM execution poses a new and practical challenge to reliable HPC-based detection. Nevertheless, it mainly focuses on quantifying detection degradation and does not investigate which microarchitectural events remain robust under such interference.

From a complementary perspective, prior work on web privacy by by Gulmezog et al. [12] has demonstrated that browser-side JavaScript execution itself can generate distinctive and measurable hardware performance signatures. Notably, the study shows that HPCs can be exploited to infer sensitive information from web workloads, underscoring the inherent complexity and noisiness of browser execution environments.

In summary, existing work has established the feasibility of HPC-based detection for Spectre and related transient execution attacks, and has highlighted the challenges posed by workload diversity, adversarial evasion, and background noise. However, the robustness of individual HPC events under realistic web-based LLM interference remains largely unexplored. In particular, it is unclear which microarchitectural features can reliably capture Spectre-induced behavior when dominant browser-side noise is present.

In contrast to prior work that primarily relies on cache-level or branch-level statistics, Spec-LAMP systematically evaluates speculation-coupled HPC events and identifies features that remain robust under Web-based LLM interference. By focusing on microarchitectural effects tightly coupled with speculative execution rather than derived cache-level outcomes, Spec-LAMP fills an important gap in existing research and enables reliable Spectre detection in modern, noisy web execution environments.

## 6. Conclusions

This paper presents the first research study on robust HPC-based Spectre detection under concurrent web-based LLM workloads. We show that noise from browser-mediated execution significantly blurs the microarchitectural footprint of speculative attacks, making conventional branch and LLC-based detector performance degradation significant in realistic web-centric settings. To address this challenge, we propose Spec-LAMP, a resilience-aware framework that identifies and integrates a critical speculation-sensitive event, *L1D miss pending*. Extensive evaluation confirms that *L1D miss pending* remains highly robust under web-induced interference and enables Spec-LAMP to restore average detection accuracy from 85.15% to 98.43% across four major Spectre variants. Our analysis further indicates that the dominant interference stems from the web execution environment itself rather than specific LLM architectures, suggesting good generalization across heterogeneous web-based LLM services.

Future work will focus on improving portability by developing adaptive feature selection strategies across diverse processor microarchitectures and on extending evaluation to a broader range of real-world noise sources, including varying browser behaviors and concurrent cloud services, to further validate Spec-LAMP’s generalizability.

## Figures and Tables

**Table 1:** Selected Cache-Related Hardware Performance Events.

Event Name	Description	Programming Info
L1D_PEND_MISS.PENDING	Measures the cumulative duration of outstanding L1D demand load misses, reflecting the number of cycles with pending fill buffer entries.	EventSel = 48H UMask = 01H
L1D.REPLACEMENT	Counts L1D cache line replacements, including both opportunistic and stall-inducing replacements.	EventSel = 51H UMask = 01H
L2_RQSTS.MISS	Counts all requests that miss the L2 cache.	EventSel = 24H UMask = 3FH
L2_RQSTS.REFERENCES	Counts all requests to the L2 cache.	EventSel = 24H UMask = FFH
L2_RQSTS.ALL_DEMAND_MISS	Counts demand requests that miss the L2 cache.	EventSel = 24H UMask = 27H
L2_RQSTS.ALL_DEMAND_REFERENCES	Counts demand requests to the L2 cache.	EventSel = 24H UMask = E7H
LONGEST_LAT_CACHE.MISS	Counts core-originated cacheable requests that miss the LLC, including data/code reads, RFOs, speculative accesses, and hardware prefetches.	EventSel = 2EH UMask = 41H
LONGEST_LAT_CACHE.REFERENCE	Counts core-originated cacheable requests to the LLC, including data/code reads, RFOs, speculative accesses, and hardware prefetches.	EventSel = 2EH UMask = 4FH

**Table 2:** Experimental Configuration and Attack Parameters.

Category	Configuration
**Hardware Configuration**	
Processor	Intel Xeon Silver 4210 (Cascade Lake)
CPU Cores/Frequency	20 cores @ 2.20 GHz
Main Memory	125.5 GiB
**Software Configuration**	
Operating System	Ubuntu 18.04.6 LTS (64-bit)
Linux Kernel	5.4.0-146-generic
HPC Collection Tool	perf 5.4.229
Programming Language	Python 3.10
IDE	PyCharm Professional 2022.1.3
Browser	Google Chrome 144.0.7559.133
Browser Automation	Selenium
**Spectre Attack Configuration**	
Evaluated Attack Variants	Spectre-PHT, Spectre-BTB, Meltdown, Spectre-SSB
Attack Implementation	Publicly available PoC with minor adaptations
Execution Mode	Standalone process, fresh invocation per sample
Attack Duration	Variant-dependent, typically <500 ms
**HPC Monitoring Configuration**	
Sampling Mode	Counting mode
Readout Interval	500 ms
Monitored HPC Events	Branches, Branch Misses, LLC References,
	LLC Misses, L1D Miss Pending
Derived Features	Branch miss rate, LLC miss rate
**Dataset Construction and Split**	
Execution Environments	Pure execution; Web-based LLM interference
LLM Services	DeepSeek, Kimi, Doubao, Qwen
Samples per Condition	600 attack + 600 benign
Conditions per Variant	1 pure + 4 LLM-interfered
Total Samples per Variant	∼6000
Training Data	Pure execution environment only
Testing Data	Web-based LLM interference only
Split Strategy	Environment-level separation (no overlap)
Class Balance	Strictly balanced (1:1)

**Table 3:** Detection Accuracy Comparison under Web-based LLM Interference.

Method	Web-LLM	Attack Variants(%)				Average (%)	Overall Average (%)
		Spectre-PHT	Spectre-BTB	Meltdown	Spectre-SSB		
baseline [8]	DeepSeek	99.3	99.42	99.3	94.55	98.14	85.15
	Kimi	93.74	86.43	87.59	81.82	87.4	
	Doubao	87.35	78.56	70.53	80.16	79.15	
	Qwen	70.42	80.71	77.65	74.76	75.89	
	None	99.73	99.73	99.91	100	99.84	\
Spec-LAMP	DeepSeek	99.65	99.46	99.88	100	99.75	98.5
	Kimi	99.77	97.22	100	100	99.25	
	Doubao	97.8	98.38	99.65	98.96	98.7	
	Qwen	95.82	92.93	96.46	100	96.3	
	None	99.91	100.00	100	100	99.98	\

**Table 4:** LOSO Evaluation of Spec-LAMP under Web-based LLM Interference.

Web-LLM	Attack Variants (%)				Average (%)	Overall Average(%)
	Spectre-PHT	Spectre-BTB	Meltdown	Spectre-SSB		
Deepseek	99.19	99.65	99.54	96.52	98.73	96.89
Kimi	99.2	98.03	98.26	93.27	97.19	
Doubao	97.22	99.65	93.62	95.59	96.52	
Qwen	93.25	99.84	93.25	94.21	95.14	

**Table 5:** Statistical Ranking of Hardware Performance Counter Events under Web-based LLM Workloads.

Event	$p_{t}$	$p_{\mathrm{ks}}$	|t|	KS	Score	Rank
L1D_PEND_MISS.PENDING	<1 × $10^{-300}$	<1 $\times 10^{-300}$	83.31	0.87	1	1
LLC_MISS_RATE	<1 $\times 10^{-300}$	<1 $\times 10^{-300}$	56.97	0.648	0.681	2
LONGEST_LAT_CACHE.MISS	<1 $\times 10^{-300}$	$5.9\times 10^{-284}$	41.24	0.464	0.456	3
L1D.REPLACEMENT	$4\times 10^{-167}$	$1.4\times 10^{-119}$	28.86	0.354	0.303	4
LONGEST_LAT_CACHE.REFERENCE	$1.2\times 10^{-163}$	$1.1\times 10^{-104}$	28.52	0.332	0.286	5
L2_RQSTS.MISS	$1.8\times 10^{-160}$	$4.2\times 10^{-101}$	28.21	0.326	0.28	6
L2_RQSTS.ALL_DEMAND_MISS	$8.3\times 10^{-159}$	$3.4\times 10^{-98}$	28.03	0.321	0.276	7
L2_RQSTS.REFERENCES	$5.7\times 10^{-92}$	$2.8\times 10^{-65}$	20.85	0.263	0.19	8
L2_RQSTS.ALL_DEMAND_REFERENCES	$2.1\times 10^{-68}$	$2.8\times 10^{-56}$	17.8	0.244	0.158	9
L2_MISS_RATE	$1\times 10^{-8}$	$4\times 10^{-19}$	5.73	0.142	0.011	10
L2_DEMAND_MISS_RATE	$3.4\times 10^{-11}$	$4.9\times 10^{-15}$	6.64	0.125	0.006	11

**Table 6:** Detection Performance of Individual HPC Features under Web-based LLM Interference.

Name	Event Count	TP	FN	TN	FP	Precision	Recall	F1	Accuracy (%)
Baseline	4	0.9084	0.0916	0.7946	0.2054	0.8157	0.9084	0.8595	85.15
L1D miss pending	5	0.9939	0.0061	0.9748	0.0252	0.9752	0.9939	0.9845	98.43
L1 replacement	5	0.814	0.186	0.9075	0.0925	0.8979	0.814	0.8539	86.07
L2 misses	5	0.8696	0.1304	0.8921	0.1079	0.8896	0.8696	0.8795	88.08
L2 references	5	0.8818	0.1182	0.879	0.121	0.8793	0.8818	0.8805	88.04
L2 demand misses	5	0.9126	0.0874	0.8458	0.1542	0.8555	0.9126	0.8831	87.92
L2 demand references	5	0.857	0.143	0.943	0.057	0.9376	0.857	0.8955	90.00

## Data Availability

The source code of Spec-LAMP is publicly available at: https://github.com/Hamster-K/Spec-LAMP (accessed on 17 January 2026).
